# Assessment of long-term psychosocial outcomes in N-methyl-D-aspartate receptor encephalitis – the SAPIENCE study protocol

**DOI:** 10.1186/s12883-024-03842-6

**Published:** 2024-09-06

**Authors:** Ole Jonas Boeken, Josephine Heine, Marta Duda-Sikula, Víctor Patricio, Géraldine Picard, Chloé Buttard, Marie Benaiteau, Álvaro Mendes, Fuchsia Howard, Ava Easton, Donata Kurpas, Jérôme Honnorat, Josep Dalmau, Carsten Finke

**Affiliations:** 1 Department of Neurology, Charité – Universitätsmedizin Berlin, Charité Campus Mitte, Charitéplatz 1, 10177 Berlin, Berlin, Germany; 2 Department of Family and Pediatric Nursing, Faculty of Health Sciences, WrocławDepartment of Family Medicine, Wroclaw Medical University, Bartla 5 St., wyb. Ludwika, Pasteura1, Wroclaw, 51-618, 50-367 Poland; 3 Fundacio de Clinic per a la Recerca Clinic Barcelona - Biomédica (FCRB) - Institut de Investigacions, Biomediques August Pi I Sunyer, c/Rosselló 149-153, Barcelona, Spain; 4 French reference center on paraneoplastic neurological diseases and autoimmune encephalitis, UMR MELIS Inserm, Université Claude Bernard Lyon1, Hôpital neurologique 59 Bd Pinel, Bron cedex, 69677, 1314 / CNRS 5284 France; 5 Instituto de Investigação e Inovação em Saúde, Universidade do Porto, Portugal, Rua Alfredo Allen, 8, 4200-180 Porto, Portugal; 6 Faculty of Applied Sciences, The University of British Columbia, T201 – 211 Westbrook Mall, Vancouver, Canada; 7 Encephalitis International, YO17 7DT Malton, UK; 8 Department of Clinical Infection, Microbiology and Immunology, University of Liverpool, Liverpool, UK

**Keywords:** NMDA receptor encephalitis, Health-related quality of life, Patient-reported outcome measures, Patient-reported experience measures, Cognitive outcomes, Post-acute interventions, Social & caregiver burden

## Abstract

**Background:**

N-methyl-D-aspartate-receptor (NMDAR) encephalitis is a rare neurological autoimmune disease with severe neuropsychiatric symptoms during the acute phase. Despite good functional neurological recovery, most patients continue to experience cognitive, psychiatric, psychological, and social impairments years after the acute phase. However, the precise nature and evolving patterns over time of these long-term consequences remain unclear, and their implications for the well-being and quality of life of predominantly young patients have yet to be thoroughly examined.

**Methods:**

SAPIENCE is a European multi-center (*n* = 3) prospective observational cohort study studying the long-term cognitive, psychiatric, psychological, and social outcome in patients with NMDAR encephalitis. The study consists of three interconnected levels. Level 1 comprises a qualitative interview and focus groups with patients and their caregivers. Level 2 consists of a condensed form of the interview, standardized questionnaires, and a detailed neuropsychological examination of patients. Level 3 involves an online survey that will be open to patients world-wide and explores patient-reported outcomes (PROMs), and patient-reported experiences (PREMs) in association with clinical and cognitive outcomes. Levels 1 to 3 will progressively contribute developing of structured interviews, survey questions, and treatment guidelines by informing one another.

**Discussion:**

SAPIENCE is an in-depth study of the long-term effects of NMDAR encephalitis and bridges the gap between standardized assessments and individual patient experiences, intending to improve patient care and to increase awareness of the psychosocial long-term consequences of the disease. Through collaboration of experts in clinical neurology and social and health psychology across Europe, SAPIENCE aims to create online assessment tools and formulate guidelines for patient-centered post-acute care that will help enhance the quality of life for patients and caregivers.

## Background

N-methyl-D-aspartate receptor (NMDAR) antibody-associated encephalitis is a rare autoimmune disorder affecting the central nervous system. It has an estimated annual incidence of 1.5 cases per million people [1] and a prevalence of 0.6 cases per 100,000 individuals [2], predominantly affecting young adults (median age = 21) [3]. During the acute phase of the disease, patients experience a range of neurological and psychiatric symptoms that can include psychosis, hallucinations, cognitive deficits, language impairment, seizures, movement disorders, autonomic instability, and disorders of consciousness [4]. Since its discovery in 2007 [5], a significant body of research has contributed to describe the clinical features of the acute phase [6–8], develop diagnostic criteria [9, 10], study neuroimaging correlates [11–15], and evaluate treatment outcomes [16–18] of the disease. In contrast, studies investigating the post-acute phase and the individual psychosocial long-term outcomes remain scarce.

Despite the severe acute disease phase, the functional neurological outcome as defined with the modified Rankin Scale (mRS) [6, 16] seems comparably favorable [19]. However, self-reports from patients in the post-acute phase indicate long-term deficits, including impaired memory functions [20, 21], reduced ability to concentrate on activities of daily living [20–22], increased fatigability [21, 23], sleep problems [24], and social withdrawal [21]. In addition to these subjective experiential reports, objective neuropsychological findings indicate long-term deficits in several cognitive areas including episodic and working memory, and executive, language and visuospatial functions in most patients .

Research conducted on small numbers of patients has revealed that persisting cognitive deficits in these individuals significantly contribute to adverse post-acute phase outcomes [20, 21, 25]. In accordance with this observation, a recent longitudinal investigation in 43 patients revealed that deficits in executive functions, episodic memory, and working memory persisted for a median of 4.3 years following the onset of symptoms, despite favorable neurological recovery (as indicated by a mRS scores of 0–1, denoting either no or only mild physical symptoms) [26]. Higher disease severity during the acute phase, longer duration of the acute stage, and delayed treatment were identified as predictors for impaired cognitive outcomes. Similarly, another recent prospective study showed that NMDAR encephalitis patients exhibit persisting cognitive and psychiatric deficits [27]. Cognitive outcomes were predicted by two acute-stage features, decreased consciousness and no improvement within the first four weeks of treatment.

Beyond the burden posed by cognitive impairments, recent research has provided initial insights into the long-term health-related quality of life outcomes for individuals with NMDAR encephalitis [28]. Psychosocial functioning among these patients was found to be lower compared to other patient groups with chronic illness and was associated with factors such as initial misdiagnosis and the low availability of follow-up psychiatric outpatient care, as revealed in a recent online survey [29]. Furthermore, lower quality of the transition from hospital care to outpatient care was linked to an increased burden on caregivers [30]. Telephone interviews conducted with 77 patients with autoimmune encephalitis revealed ongoing fatigue, short-term memory deficits, emotional instability, and concentration problems [31]. Indeed, fatigue as well as sleep disturbances are increasingly reported following NMDAR encephalitis [21, 24, 32, 33]. Recently, it was observed in a group of 22 patients that persisting neuropsychiatric symptoms were associated with reduced social and global quality of life [34]. Overall, neuropsychiatric sequelae can persist for years beyond the post-acute phase, impacting not only the patients’ lives and their capacity to return to work or education but also imposing a substantial burden on caregivers and close relatives [29, 30, 35].

However, data from previous studies on psychosocial function is limited due to small sample sizes, mixed samples of different autoimmune encephalitis syndromes and isolated analyses of potential determinants of quality of life [28, 36]. There is a lack of comprehensive, in-depth investigations into the long-term psychosocial impact of the disease, especially in larger patient cohorts, despite the relatively young age of the patients and the negative impact on their quality of life. It is therefore essential that such studies include the perspective of patients, relatives and caregivers [28]. Moreover, there are currently no diagnostic or treatment guidelines available for the post-acute phase of NMDAR encephalitis that could be informed by the results of these studies.

SAPIENCE (Social and psychological long-term impact of NMDA receptor encephalitis) represents the inaugural effort to connect standardized assessments with the unique experiences of individual patients with autoimmune encephalitis in the extended follow-up. Based on an interdisciplinary and interprofessional team and through the inclusion of patient and caregiver perspectives, SAPIENCE aims to create online assessment tools and to establish treatment guidelines tailored to patient-centered post-acute care that will help to improve the quality of life for both patients and caregivers.

## Methods

### Objectives

SAPIENCE addresses the cognitive, psychiatric, psychological, and social consequences of NMDAR encephalitis in large and multi-national cohorts at three study sites: Berlin (Germany), Barcelona (Spain) and Lyon (France). The project aims to improve the treatment of patients with NMDAR encephalitis by (1) providing a comprehensive assessment of long-term psychosocial outcomes, (2) describing the patient journey after the acute phase, and (3) examining how outcomes differ by patient, culture, and health care system. The desired outcome is to generate patient-centered guidelines for the post-acute care of individuals with this rare disease. We will also construct a framework to capture disease-related experiences and challenges and identify the factors that influence long-term subjective outcomes in these patients.

### Design

Our approach employs a three-tiered study design, incorporating qualitative (level 1), mixed-methods (level 2), and quantitative (level 3) methodologies. The three-level study design enables the synthesis of the strengths of qualitative and quantitative methodologies [37].

### Level 1: Qualitative semi-structured interviews & focus groups

Level 1 examines the personal challenges, symptoms, and access to medical care experienced by patients with NMDAR encephalitis and their caregivers. Here, five patients and their caregivers will be invited for individual semi-structured interviews about their personal experience with the disease and treatment at each study site. Furthermore, a focus group with a different set of patients and their caregivers (*n* = 10, i.e., five patients and their caregivers, for each site) will be conducted at each site (on-site or hybrid-online) to gain a deeper understanding of joint perspectives and experiences of the patient journey. Specifically, we employ a qualitative interpretive descriptive approach [38] that allows for the identification of patterns and themes representing subjective perspectives. Both parts include open-ended questions, follow a topical interview guide, and will be audio-recorded and supplemented with field notes. We will code the data based on verbatim transcripts to identify categories and apply concept mapping to visualize their relationships [39].

In addition to characterizing psychosocial outcomes in a large sample of NMDAR encephalitis patients, a primary objective of this project is to create structured interviews and questionnaires tailored for clinical use in the context of this disease. We will employ comprehensive qualitative methods to ensure that these tools accurately capture patients’ experiences and address their specific needs. These insights will play a significant role in study’s subsequent mixed-methods and quantitative phases, which encompass levels 2 and 3. Hence, the framework established during the interviews and focus groups at level 1 will be applied to generate a concise, on-site version of the semi-structured interview, to capture patient experiences in a broader and more diverse sample across all study sites.

### Level 2: Mixed-methods approach – comprehensive cognitive and psychosocial assessments

In Level 2, qualitative methodologies are integrated with thorough quantitative cognitive evaluations and questionnaire assessments to provide a comprehensive, long-term profile of NMDAR encephalitis in *N* = 90 patients (i.e., 30 per site). Clinician-observed metrics (such as neuropsychological test outcomes and disability assessments) will be juxtaposed with patient-reported results (including quality of life, metacognition, and subjective evaluations of physical and psychosocial functioning) with the aim of pinpointing disparities and, in the end, uncovering gaps in healthcare. NMDAR encephalitis is a rare disease, meaning that the available pool of patients is limited. Consequently, we will implement an online administration for the neuropsychological test battery and questionnaires to achieve the desired recruitment targets.

The neuropsychological assessment will involve language-adapted tests, i.e. tests will be tailored each study site’s normalization and language requirements. These tests will be utilized to evaluate executive functions (i.e., verbal fluency [40] and working memory via digit span backward [41, 42]), long-term memory retention and retrieval (i.e., Rey-Auditory Verbal Learning Test or RAVLT [43]), short-term memory (i.e., digit span forward [41, 42]), and spatial navigation (i.e., the Virtual Environment Navigation Assessment or VIENNA ) [44]. These cognitive domains are often impaired in NMDAR encephalitis [26]. Verbal tests are conducted by videoconference which has proven reliable [45]. The spatial navigation test, VIENNA, is administered in a web-based format [44].

Subjective experiential reports will be gathered using the psychosocial patient-reported outcome measures (PROMS) Field, (PROMs) [46], patient-reported experience measures (PREMS) Field [47, 48], and meta memory [49, 50] .The selected PROMs record the domains of fatigue (Fatigue Severity Scale, FSS [51]), sleep (Pittsburgh Sleep Quality Index, PSQI [52]), self-assessment of memory performance (Multifactorial Memory Questionnaire, MMQ [49, 50]), depression (Beck Depression Inventory, BDI-II [53], anxiety (Hospital Anxiety and Depression Scale, HADS [54]), and patients’ quality of life (5-Level EQ-5D, EQ-5D-5 L [55]). These factors have been shown to significantly influence patients’ post-acute state of health and overall global health [26, 27, 36, 56].

PREMS assess patients’ encounters and requirements throughout their care journey [48, 57]. In particular, our research will evaluate how patients perceive interactions with healthcare providers, the consistency of their treatment, the shared decision-making processes, the participation of their caregivers, and the physical and emotional support they receive during their treatment. This patient perspective will be investigated using a survey tool developed based on the qualitative analysis of Level 1 semi-structured interviews. This approach addresses the need for standardized multilingual PREMs available for all study site languages (i.e., French, German, and Catalan/Spanish) that would require translations and validations at local sites. Since PREMs are typically language-specific and culturally embedded, reflecting different healthcare systems [48, 58], our survey aims to provide a bias-free assessment by directly considering the diverse backgrounds of our patients.

Finally, we will assess the role of the following potential outcome predictors: (i) disease duration/severity (mRS, Clinical Assessment Scale (CASE [59])); (ii) intensive care treatment; (iii) difficulties during treatment (e.g., delayed diagnosis, relapse); (iv) availability and duration of rehabilitation; (v) pre-existing comorbidities; and (vi) socioeconomic background.

### Level 3: Online network-level assessment of quality of life and healthcare experiences

Level 3, an online study, will invite the participation of patients worldwide and allow assessment of the impact of different healthcare systems and cultural factors on outcomes. Thus, this study level is designed to be independent of site-specific factors and suitable for a broad range of patients with NMDAR encephalitis. For instance, the study will consider that access to specialists and availability of treatment options differ between countries.

Cognitive assessments will center on self-reported measures, including the MMQ [49] and VIENNA [44], for analysis of spatial navigation, a function highly dependent on the integrity of the hippocampus – a brain structure with increased vulnerability in NMDAR encephalitis. While psychosocial evaluations will rely on PROM questionnaires, PREMs will be assessed based on Level 2 short interview analysis. Thus, insights from the short-form interviews will guide the formulation of survey questions targeting pre-disease situations, disease progression, and patient experiences (e.g., interactions with healthcare professionals, treatment-related experiences, the flow of information).

The objectives of level 3 are to expand the assessment across various healthcare systems, cultural contexts, and diverse populations, and establish an online evaluation tool for pertinent outcome measures for future research because standardized interviews or surveys specifically for autoimmune encephalitis are currently unavailable. These patient-centered survey questions will be developed based on the insights gained from levels 1 and 2. They can be applied to broad and international patient populations, making them readily accessible for clinical and research purposes. Finally, the results from studies at levels 2 and 3 will be used to develop international guidelines for long-term care.

### Setting

SAPIENCE aims to characterize the long-term cognitive and psychosocial consequences of NMDAR encephalitis in an international collaboration across four European study sites. Additionally, research groups at the University of British Columbia (Canada) and the University of Porto in Portugal will contribute their expertise in qualitative research as SAPIENCE collaborators.

A central component of SAPIENCE is the close collaboration with patient advocacy groups (PAOs), including Encephalitis International (UK), Autoimmune Encephalitis Alliance (USA), Autoimmunenzephalitis Selbsthilfegruppe (Germany), and the Anti-NMDA-Rezeptor- Enzephalitis Forum (Germany). These PAOs are involved in several critical aspects of the study, including co-designing the study, defining relevant outcome measures, helping create informational materials, recruiting participants, interpreting study results, and formulating long-term care guidelines to enhance rehabilitation and life quality.

### Recruitment and eligibility criteria

Participants will be recruited from non-overlapping cohorts throughout Germany for the study site Berlin, throughout France for participation in Lyon, and throughout Spain for the study site Barcelona (nationwide recruitment). The German Network for Research on Autoimmune Encephalitis (GENERATE) will facilitate recruitment in Germany. For the Level 3 online study, patients will be invited globally to participate in this cross-sectional online analysis through study sites in Berlin, Barcelona and Lyon, as well as through participating PAOs from Germany, the UK, the USA and Canada with their global reach. Inclusion criteria for participation in the study are a confirmed diagnosis of NMDAR encephalitis [9], being over the age of 17 years, and demonstrating proficiency in one of the project languages (German, French, Spanish, Catalan, English). Exclusion criteria entail the presence of preexisting neurological disorders and the inability to provide informed consent.

### Hypotheses

The main hypothesis is that NMDAR encephalitis is associated with a long-lasting and clinically relevant impact on patient’s cognitive, psychiatric, psychological, and social well-being (H1). Cognitive deficits such as memory impairment and deficits of executive functions are persistent in many patients and impact the return to work/school and independence in daily activities (H1.2). Fatigue, sleep quality, affective symptoms, and cognitive complaints are major determinants of the subjective long-term outcome of NMDAR encephalitis (H1.3). Post-acute symptom severity and quality of life are related to characteristics of the acute disease course (e.g., treatment delay, need for intensive care unit stay), access to post-acute care, caregiver support, and personal coping strategies (H1.4). Structured interviews developed in this study will inform the development of patient-centered guidelines for long-term care and rehabilitation strategies (H1.5).

### Sample size

The sample size of *N* = 30 for the semi-structured interviews and focus groups in Level 1 is based on current recommendations for investigating of clinically relevant participant characteristics and the derivation of conceptual categories [60]. The sample size of *n* = 90 for Level 2 was calculated to detect medium effect sizes in correlation analyses (power 80%, significance level 5%, correlation coefficients of > = 0.23; R package pwr [61]). Calculation of the sample size for the online study (Level 3) was based on an estimated prevalence of 50% of patients with impaired health-related quality of life and assuming an estimated population size of *N* = 500 in Germany (as an example), a confidence level of 1- α = 0.95, and a margin of error of e = 5%. The sample size is calculated to be *n* = 218 based on the formula n=[(z2p(1-p))/e2]/[1+(z2p(1-p))/e2*N]. Considering an estimated 30% missing data during the survey, we plan for an *N* = 300 for the online survey.

### Statistical analyses

Statistical analyses encompass a multifaceted approach. Firstly, cognitive deficits will be analyzed using test-specific norm values and patient-reported outcomes measures will be evaluated utilizing established normative data. Furthermore, correlation analyses will be used to explore the relationships between cognitive deficits and clinical parameters, including symptom severity and duration of hospitalization. In addition, correlation analyses with patient-reported outcomes, encompassing quality of life, affective symptomatology, physical impairment, and self-assessed memory function will be studied. Lastly, regression analyses are performed to identify predictors of quality of life.

## Discussion

SAPIENCE is a prospective, interdisciplinary, multi-center study to investigate the long-term cognitive, psychiatric, psychological, and social repercussions faced by patients with NMDAR encephalitis. The psychological and social consequences of NMDAR encephalitis have significant impacts on patients’ well-being - yet they remain inadequately understood, frequently undetected, and untreated. SAPIENCE aims to bring about a substantial shift in the care of individuals with NMDAR encephalitis by (1) delivering a comprehensive assessment of long-term psychosocial effects associated with the condition, (2) delineating the patient’s experience during the post-acute phase, and (3) investigating the variability of outcomes among patients, considering cultural and healthcare system differences. The aim if SAPIENCE is to create patient-focused treatment recommendations that can be adopted globally by medical practitioners and healthcare providers. As a result, we anticipate substantial progress in understanding the disease, improved health outcomes for both patients and caregivers, enhanced assessment tools such as novel disease-specific interviews and surveys, and potential economic benefits such as reduced healthcare expenses.

SAPIENCE will need to address the challenges inherent to the international multi-center design of the study in a rare disease. The international multi-center approach that relies on a close interdisciplinary collaboration between clinical teams, Social Sciences and Humanities (SSH)-experts and patient advocacy organizations requires a robust project governance. This includes defining responsibilities, stakeholder communication, risk management, ethical compliance, data protection, and continuous project status monitoring. Furthermore, reliable and standardized data acquisition and analysis processes must be established and harmonized across study sites. This will entail the development of standard operating procedures and manuals for the semi-structured interviews and neuropsychological tests, training of the experimenters involved in data acquisition at each study site using online seminars, and regular peer-to-peer and supervision meetings led by team members with relevant qualitative and neuropsychological expertise.

In this three-level study design, we will use existing infrastructure and patient registries to enrol and evaluate patients across three research sites. Data collection on-site for levels 1 and 2 is scheduled to take place at three locations: Charité - Universitätsmedizin Berlin, the French Reference Center on Paraneoplastic Neurological Diseases and Autoimmune Encephalitis in Lyon, and the Fundació de Recerca Clínic Barcelona. Given the rarity of the disease, a significant challenge will be the recruitment of a sufficiently large sample of patients with NMDAR encephalitis. For Level 1, this pertains to the recruitment of patient and caregiver dyads while for Levels 2 & 3 this relates to sample size goals of *n* = 90 and *n* = 300, respectively. To improve recruitment, the SAPIENCE team will closely collaborate with patient advocacy organizations and leverage existing infrastructure such as the GENERATE network. To allow for robust cross-site analyses, SAPIENCE will use only standardized neuropsychological tests and questionnaires available in the languages of the three project sites and with available normative data.

Critical aspects for the Level 3 (online study) include (i) the establishment of data storage and data protection procedures that meet the requirements and regulations of all participating countries; and (ii) the creation of a suitable informed consent process that adheres to the legal and ethical standards of all participating countries. In addition, potential biases inherent to online studies must be considered and mitigated. Such biases include the possibility of participants submitting duplicate responses, individuals responding inattentively, and the possible bias of preferentially recruiting technology-savvy responders. To address these biases, we have devised several measures. To counteract duplicate responses, participants will be required to generate a unique, non-identifiable code that remains consistent. This code will identify and remove duplicate entries from our dataset. To address inattentive responding, we will employ various techniques. Survey tools will incorporate time-bias scoring to identify and flag participants who rush through the survey. Additionally, attention-check items and questions will be strategically placed throughout the study to ensure that participants are actively engaged and provide thoughtful responses. For participants needing more time to be comfortable with online questionnaires, we will offer a pen-and-paper version as an alternative. The data collected via this method will then be manually entered into our system study team member.

In conclusion, SAPIENCE will be an interdisciplinary and international study aiming to improve the long-term the cognitive, psychiatric, psychological, and social outcomes of patients with NMDAR encephalitis by combining objective and subjective measures and integrating the perspectives of healthcare providers, patients, and caregivers resulting in patient-centered post-acute care treatment guidelines.

## Data Availability

No datasets were generated or analysed during the current study.

## Abbreviations

EQ-5D-5L: 5-Level EQ-5D
BDI-II: Beck’s Depression Inventory
CASE: Clinical Assessment Scale
FSS: Fatigue Severity Scale
GENERATE: German Network for Research on Autoimmune Encephalitis
HADS: Hospital Anxiety and Depression Scale
mRS: modified Ranking Scale
MMQ: Multifactorial Memory Questionnaire
NMDAR: N-methyl-D-aspartate receptor
PAOS: Patient Advocacy Organizations
PREMS: Patient-Reported Experience Measures
PROMS: Patient-Reported Outcome Measures
PSQI: Pittsburgh Sleep Quality Index
RAVLT: Rey-Auditory Verbal Learning Test
SAPIENCE: Social and psychological long-term impact of NMDA receptor encephalitis
VIENNA: Virtual Environment Navigation Assessment

## Declarations

Levels 1 and 2 of SAPIENCE have been granted ethical approval at all study sites (Ethikkommission der Charité – Universitätsmedizin Berlin (EA2/138/23, 13.07.2023); Comité Ético de Investigación Clínica del Hospital Clínic de Barcelona; Comité de Protection des Personnes EST III (2023-A01446-39; NCT05954468)).

Approvals for Level 3 are currently underway in Berlin and Lyon, with ethical approval already granted for the Spanish site.

The study is being conducted under the Declaration of Helsinki and Good Clinical Practice guidelines, which include the protection of data and patient privacy. All participants are required to provide written informed consent before participating.
